# Correction: Gas-phase synthesis of the bicyclic silicon tricarbide molecule (c-SiC_3_) as a precursor to silicon carbide nanoparticles in space

**DOI:** 10.1039/d6sc90061e

**Published:** 2026-03-09

**Authors:** Shane J. Goettl, Kazuumi Fujioka, Márcio O. Alves, Mateus X. Silva, Zhenghai Yang, Surajit Metya, Iakov A. Medvedkov, Tosaporn Sattasathuchana, Breno R. L. Galvão, Rui Sun, Ralf I. Kaiser

**Affiliations:** a Department of Chemistry, University of Hawai'i at Mānoa Honolulu HI 96822 USA ralfk@hawaii.edu ruisun@hawaii.edu tsatta@hawaii.edu; b Centro Federal de Educação Tecnológica de Minas Gerais Belo Horizonte 30421-169 Brazil brenogalvao@gmail.com

## Abstract

Correction for ‘Gas-phase synthesis of the bicyclic silicon tricarbide molecule (c-SiC_3_) as a precursor to silicon carbide nanoparticles in space’ by Shane J. Goettl *et al.*, *Chem. Sci.*, 2026, https://doi.org/10.1039/d6sc00002a.

The authors regret that a footnote indicating that the first two authors, Shane J. Goettl and Kazuumi Fujioka, contributed to this work equally was omitted from the original paper.

The Royal Society of Chemistry apologises for these errors and any consequent inconvenience to authors and readers.

